# Legal Notes for Institutional Workers

**Published:** 1911-10-21

**Authors:** 


### LEGAL NOTES FOR INSTITUTIONAL WORKERS.

(The Editor will be pleased to answer legal queries in this column.)

### A POPULAR DELUSION.

In some minds there appears to be a rooted belief that the doctor prefers to work for nothing rather than not work at all. In a case which recently came within the writer's own experience at the Huntingdon County Court, it appeared that the child of a labourer was suffering from scarlet fever. A doctor who was in attendance suggested that the patient should be removed to the isolation hospital where free nursing was provided. He continued to attend the patient at the hospital, and finally sent in his bill, which amounted to £1 1s. for twelve visits. The parents refused to pay, saying that the doctor had stated the removal would occasion no expense. The doctor having denied making any such statement, the County Court judge found in his favour, ordering the defendant to pay 2s. a month. He could not have decided otherwise. But the case has its moral. When sending a patient to an isolation hospital the practitioner should make it quite plain that if he is to continue to attend he must be paid his fees.

### HOSPITALS AND PATENTS.

The patent agent who takes note of what is going forward in the hospital must often wonder why his services are not invoked with greater frequency in relation to medical appliances and apparatus. "Novelty" and "utility"—those essential attributes of a patentable invention—are the features of many new surgical instruments. What a fortune Murphy would have made if he had obtained a patent for his "button"! The explanation appears to be that the medical profession has by general consent decided to give to the public the benefit of all its mechanical ingenuity. The writer is acquainted with a distinguished surgeon whose skill at the operating table is only equalled by dexterity with lathe and file in his workshop. He devised a modification of some surgical instrument which was both new and useful, and made a few specimens for his own use. An astute German instrument maker, having got hold of the idea, proposed to make it the subject-matter of letters patent, on half-share terms. Not only did the surgeon refuse the offer, but he immediately wrote to one of the medical papers, giving a minute description of the instrument, showing how it was made and what it was for. This publication effectually prevented the grant of a valid patent.

### SOME HINTS TO THE MEDICAL WITNESS.

One of the incidents of an "appointment"—whether salaried or otherwise—is liability to give evidence in courts of justice. To answer questions, even on oath, may appear to be a simple matter; but the junior house surgeon generally looks forward to his first appearance in court with some degree of apprehension. To begin with he should be careful to refuse to go to court at all in a civil case, as distinct from a criminal case, unless a fee is paid with the subpœna. Reluctance or refusal to pay this fee tends to show that the case is not bonâ fide. In a criminal case, where the witness appears for the Crown, the fees, though small in amount, are always paid. In preparing himself for the ordeal of the witness-box, the practitioner should remember that he will be allowed to refresh his memory with any notes made at the time. Thus, if he was called upon to examine a patient, and he made any entries of the symptoms in his diary, he would be entitled to refer to that diary in court in order to refresh his memory. Needless to say, such testimony carries considerable weight; but an account of the patient's condition written a long time after the consultation could not be used. Perhaps the most irritating feature of court work is the waste of time it involves. Yet experience may enable a man to save much time. Thus the judge will often release the medical witnesses until a certain hour, or fix a special day for a medical case, or take the medical witness out of his turn. The doctor who is in a hurry to be off should not hesitate to ask counsel or solicitor to make a special application to the court.

### LIABILITIES OF HOSPITAL AUTHORITIES.

The case of Gregory *v.* Torquay Corporation, which is now fully reported, incidentally raises a question of hospital authorities. The action was brought to recover damages for alleged negligent treatment at the Torquay Corporation Isolation Hospital. It was said that while suffering from the effects of scarlatina, for the treatment of which disease he had been sent to the hospital, the plaintiff's son had been allowed to do work which injured his health, causing heart disease, kidney disease, rheumatic fever. He eventually died. The defendants denied negligence, and also pleaded the Statute of Limitations. A jury found that there had been negligence, and awarded the plaintiff £50 for the death of his son, but the Divisional Court held that as the alleged act of negligence took place more than six months before the action was brought the action was void. This sounds rather extraordinary, but the explanation is simple. Local authorities are protected from actions for damages in respect of wrongful acts done in the performance of any statutory duty unless the action is brought within six months. This is the result of a statute known as the Public Authorities' Protection Act. If a Corporation in the circumstances of this case were held entitled to protection, why should not an ordinary hospital be held similarly entitled? The Act, which confers certain other privileges which need not be here referred to, extends to "the execution or intended execution of any Act of Parliament, or of any public body or authority." It has been limited, unfortunately, to statutory bodies, but it is difficult to see why a local body in its capacity as hospital owner should enjoy privileges not vouchsafed to the ordinary institution.

